# Silver Nanoparticles-Decorated Titanium Oxynitride Nanotube Arrays for Enhanced Solar Fuel Generation

**DOI:** 10.1038/s41598-017-02124-1

**Published:** 2017-05-15

**Authors:** Khaled A. Soliman, Abdallah F. Zedan, Ahmed Khalifa, Hany A. El-Sayed, Amina S. Aljaber, Siham Y. AlQaradawi, Nageh K. Allam

**Affiliations:** 1 Department of Chemistry and Earth Sciences, Qatar University, Doha, 2713 Qatar; 2 Energy Materials Laboratory (EML), School of Sciences and Engineering, The American University in Cairo, New Cairo, 11835 Egypt; 3 Department of Chemistry, Technische Universität München, Lichtenbergstrasse 4, 85748 Garching, Germany; 4 Physical Chemistry Department, National Research Centre, Dokki, Cairo 12441 Egypt; 5 National Institute of Laser Enhanced Science, Cairo University, Giza, 12613 Egypt

## Abstract

We demonstrate, for the first time, the synthesis of highly ordered titanium oxynitride nanotube arrays sensitized with Ag nanoparticles (Ag/TiON) as an attractive class of materials for visible-light-driven water splitting. The nanostructure topology of TiO_2_, TiON and Ag/TiON was investigated using FESEM and TEM. The X-ray photoelectron spectroscopy (XPS) and the energy dispersive X-ray spectroscopy (EDS) analyses confirm the formation of the oxynitride structure. Upon their use to split water photoelectrochemically under AM 1.5 G illumination (100 mW/cm^2^, 0.1 M KOH), the titanium oxynitride nanotube array films showed significant increase in the photocurrent (6 mA/cm^2^) compared to the TiO_2_ nanotubes counterpart (0.15 mA/cm^2^). Moreover, decorating the TiON nanotubes with Ag nanoparticles (13 ± 2 nm in size) resulted in exceptionally high photocurrent reaching 14 mA/cm^2^ at 1.0 V_SCE_. This enhancement in the photocurrent is related to the synergistic effects of Ag decoration, nitrogen doping, and the unique structural properties of the fabricated nanotube arrays.

## Introduction

Over the past few decades, metal oxides have been extensively explored as photoelectrodes for solar-driven production of fuel due to their exceptional stability, semiconducting properties, abundance, and low cost^1–8^. However, most metal oxides have absorption activity that is limited to the ultraviolet spectral region because of their wide band gap (>3.0 eV). This is inconvenience because the ultraviolet spectral region contains only 3–5% of all incident solar energy. Besides, metal oxides with narrow band gaps (<3.0 eV), such as Fe_2_O_3_ and WO_3_, have stability concerns or improper band alignment for water splitting and require a large external bias^2^.

An alternative class of solar energy conversion materials is transition metal oxynitrides. Since metal-nitrogen bond has higher potential energy than metal-oxygen bond, oxynitrides^9–16^ have narrower band gap energies compared to their metal oxides counterparts. Additionally, oxynitrides are stable in alkaline media making them ideal water splitting photoelectrodes^9–12^. Particularly, titanium oxynitride (TiON) is a promising material for visible light absorbtion and appropriate band-edge positions for water splitting^13–16^. Vitiello *et al*.^14^ used NH_3_ nitridation to fabricate TiON nanotube arrays from anodized Ti foil. Their TiON showed enhanced photoelectrochemical properties and significant visible light response. Efficient nanostructured mesoporous TiON thin films were reportred by Ferrero *et al*.^15^. The films resulted in a shift of the titania absorption edge, due to the introduction of N atoms. Alternatively, Kim *et al*.^16^ used Ti-N substrates to fabricate Ti-O-N nanotubes *via* anodization, resulting in a significant visible light photoresponse. Asahi *et al*.^17^ reported that N-doped TiO_2_ has an influence on the photocatalytic activity for the decomposition of acetaldehyde and methylene blue at wavelengthes up to 550 nm. Recently, Gebauer *et al*.^18^ have investigated the oxygn reduction reaction (ORR) on N-doped titanium dioxide. It was found that N-doped titanium oxide significantly improve the ORR performance compared to non-doped TiO_2_^18^. Decorating TiON materials with nanoparticles and/or sensitizers^19, 20^ has also been recently proposed as an effective method to enhance the surface catalytic activity of a plethora of materials^21–24^. Hiroaki *et al*.^22^ have examined the effect of Ag nanoparticles (NPs)-decorated TiO_2_ nanotube arrays. This hybrid device resulted in higher photocatalytic activity and solar energy conversion efficiency (~3.5 µA) compared to bare TiO_2_ electrode ((~0.5 µA)^22–24^. This enhanced catalytic effect was related to the formation of hydroxyl radicals, which were made possible through better charge-transfer processes^25, 26^.

However, most of the reported TiON materials are in the form of either powders or thin films, which are not practical for a scalable photolysis process. With thin films, the light absorption and carrier collection are in competition, i.e., although thick films are needed to harvest a reasonable amount of the solar spectrum, thicker films than the carrier diffusion length (usually tens of nanometers) will result in poor carrier collection efficiency.

It was then interesting to combine both advantages of light absorption and low overpotential catalytic activity. Herein, we report for the first time, the fabrication of silver NPs-decorated titanium oxynitride (Ag/TiON) nanotube arrays to investigate their performance as photoanodes in photoelectrochemical water splitting cells.

## Materials and Methods

Titanium foil (0.25 mm thick, purity 99.8%) was polished into portions and cleaned in acetone, ethanol and deionized water, respectively. Two- electrode electrochemical cell was used for anodization in which the Ti metal foil (positive electrode) and a platinum foil (negative electrode) were connected to a DC power supply at 30 V. Ethylene glycol-based solutions containing 0.5 M NH_4_F and 3 ml H_2_O. All samples were anodized for 120 minutes at room temperature. The resulting titanium oxide nanotubes were then annealed in ammonia flow (200 sccm) at 600 °C for two hours. The heating and cooling rates were as low as 2 °C/min to preserve the nanotubular architecture and avoid their detachment. As a reference, titanium oxide nanotubes sample was annealed in air (450 °C and 2 °C/min for 2 hours). The Ag nanoparticles were prepared by the borohydride reduction method. A 100 ml of 1 mM AgNO_3_ (Sigma-Aldrich, 99,999%) was added to a mixture of 30 ml of 2 mM NaBH_4_ (Sigma-Aldrich, 98%) under vigorous stirring. For the preparation of Ag decorated TiON nanotube arrays, 50 µl of Ag colloidal solution (the loading density is 3.42 × 10^11^ Ag NPs per 1 cm^2^ foil) was drop-casted onto the TiON foil and left to dry overnight.

Scanning electron microscopy (SEM) images and energy dispersive X-ray spectroscopy (EDX) measurements were carried out using an FEI electron scanning microscope. The powder X-ray diffraction (XRD) measurements were carried out at room temperature using Rigaku Miniflex II diffractometer with Cu KαR radiation at 30 kV and 20 mA between 2θ angles of 20 and 80° with scanning rate of 0.025° per step per second. X-ray photoelectron spectroscopy (XPS) measurements were carried out on Kratos Axis Ultra XPS with a monochromatic Al Kα radiation source (1486.6 eV) in a UHV environment (ca. 5 × 10^−9^ Torr). Transmission electron microscopy (TEM) images were acquired by an FEI Philips Technai 20 transmission electron microscope with an accelerated voltage of 200 kV. The optical absorption of the samples was measured using a Cary 5000 UV-Vis-NIR spectrophotometer. The *J*-*V* measurements were carried in a three-electrode electrochemical cell with a saturated calomel electrode (SCE), a platinum wire and the tested sample were used as reference, counter, and working electrodes, respectively. The area of the working electrode was 0.88 cm^2^ and that of the counter electrode was 3.145 cm^2^. The working electrode was immersed in 0.1 M KOH (Carl-Roth, Germany 99.98%). The KOH solution was prepared from ultrapure water (18.2 MΩ cm at 25 °C, TOC < 1 ppb) and was purged with nitrogen gas during the measurement. A scanning potentiostat (Gamry 3000) was used to measure dark and illuminated currents at a scan rate of 10 mV/s. A 100 W ozone-free xenon lamp (Abet Technologies, USA) was used as the light source, with an AM 1.5 G filter to simulate sunlight at 100 mW/cm^2^.

## Results and Discussion

**Figure 1:**
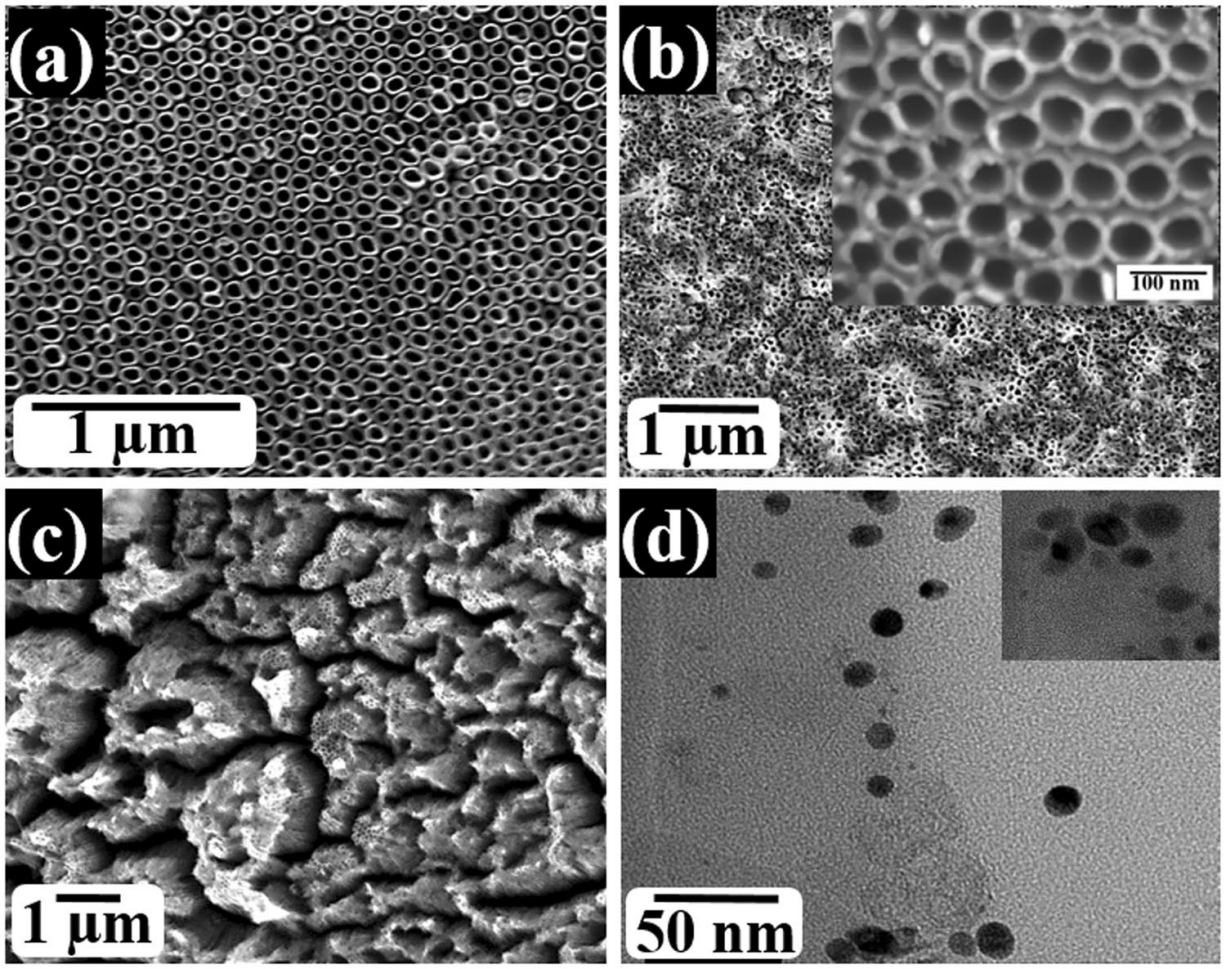
SEM images of (**a**) air-annealed, (**b**) ammonia-annealed, (**c**) Ag-decorated ammonia-annealed titanium oxide nanotube arrays, and (**d**) TEM image of the Ag nanoparticles deposited on carbon-coated copper grid.

Figure 1a shows an FESEM top-view image of the fabricated titanium oxide nanotubes. The well-aligned, densely packed nanotube arrays that are several microns long were formed and distributed uniformly with full coverage on the film surface. The average length of the nanotubes is estimated as 11.2 ± 3 µm, while the inner diameter and the wall thickness are 50 and 15 nm, respectively. The morphology and structure of the nanotubes are preserved even after annealing in air (Fig. 1a) or ammonia ambient for 2 h (Fig. 1b). Note that the nanotubular structure has not been affected by annealing, where the diameter is slightly increased into 54 nm and the wall thickness is 14 ± 2 nm. Figure 1c shows the Ag nanoparticles-decorated nanotubes. Also, Fig. 1d shows HRTEM image of the silver nanoparticles. To prepare such electrodes, 50 µL of Ag colloidal solution was drop casted onto the TiON foil and left to dry overnight. Then, the Ag/TiON surface was washed with ultrapure water. Note that the Ag nanoparticles are well-dispersed on the TEM grid with uniform size (13 ± 2 nm) and spherical shape.

**Figure 2:**
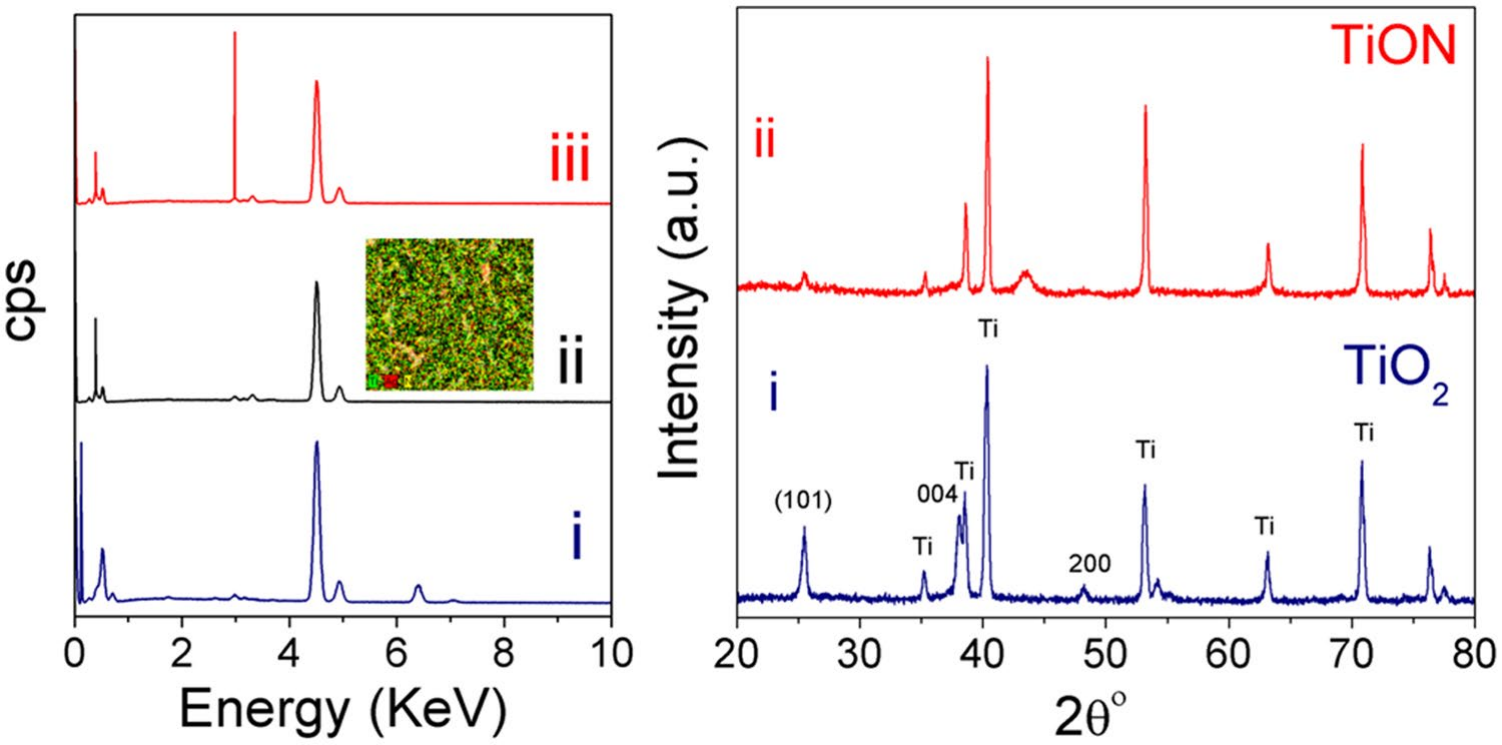
(**a**) EDX and (**b**) GAXRD spectra of (i) air-annealed, (ii) ammonia-annealed, and (iii) Ag-decorated ammonia-annealed samples. The inset in Fig. 2a is the EDS mapping for Ag nanoparticles.

**Figure 3:**
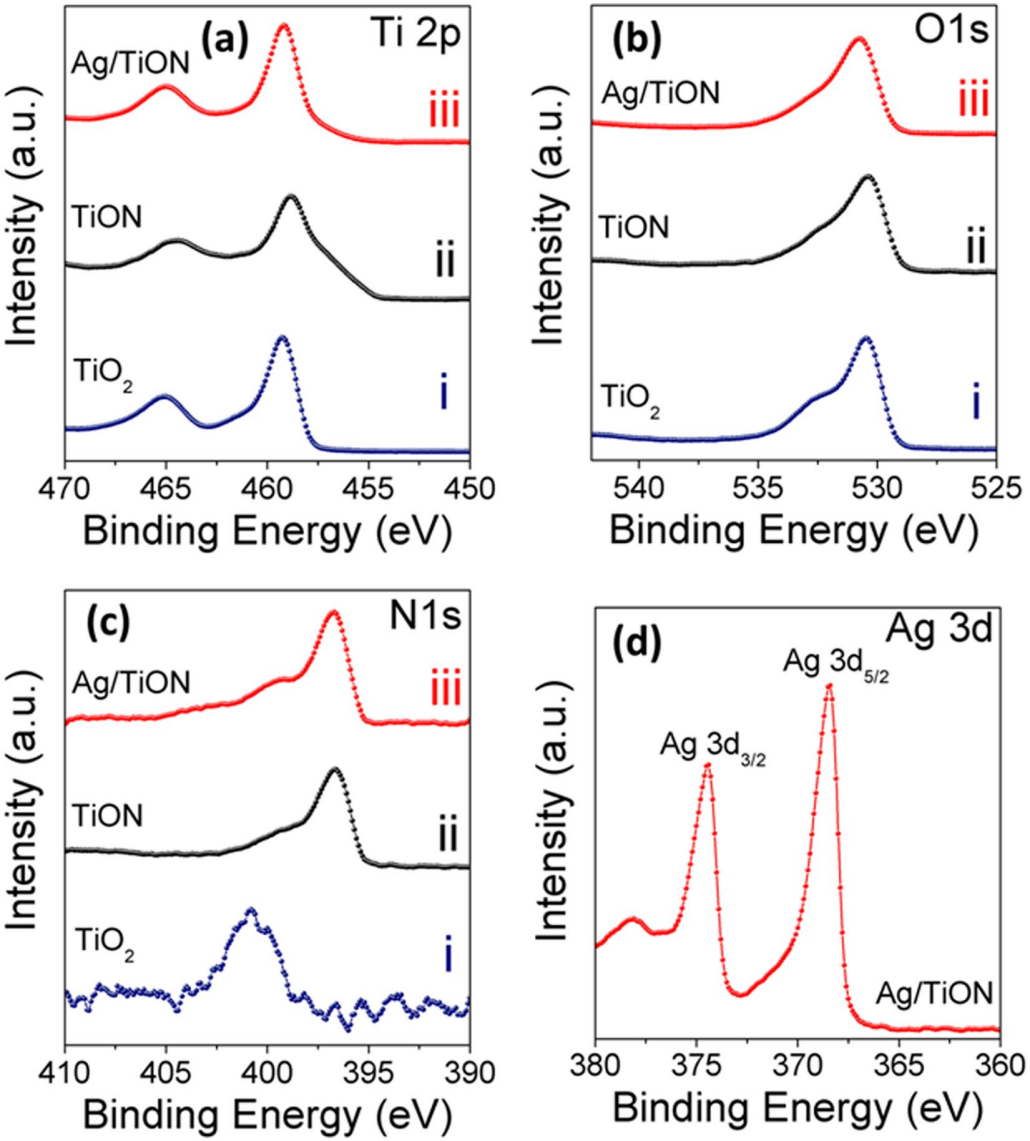
XPS spectra of the (i) air-annealed, (ii) ammonia-annealed, and (iii) Ag-decorated ammonia-annealed nanotube samples.

To investigate the structure and composition of the fabricated nanotube arrays, EDX (Fig. 2a), XRD (Fig. 2b) and XPS (Fig. 3) analyses were performed. Figure 2a shows the EDX spectra for (i) air-annealed, (ii) ammonia-annealed, and (iii) Ag-decorated ammonia-annealed titanium dioxide nanotube arrays. The peak at 0.277 eV is related to carbon species, whereas the peak at 0.525 eV is related to oxygen species. Note that the intensity of the peak at 0.525 eV decreased after annealing in ammonia and another peak emerged at 0.392 eV, which is assigned to nitrogen atoms. The sharp peak at 2.984 eV is a good indication for Ag decoration on TiON nanotube arrays. The common peak around 4.508 eV belongs to titanium species. Figure 2b shows the XRD patterns of the nanotubes annealed in air and those annealed in ammonia, revealing crystalline structures of titanium oxide. The appearance of the characteristic diffraction peaks at 25°, 38.1°, 47.8°, 52.8°, and 53.9°, corresponding to the (101), (004), (200), (105), and (211) facets, respectively elucidate the crystalline structures of titanium oxide^14^. Note that the peak at 2θ ~43° appeared in the XRD spectra of NH_3_-annealed sample corresponds to the cubic phase of titanium oxynitride as reported by Zukalova *et al*.^27^. Furthermore, the signature of the underlying Ti metal is apparent as indicated from the sharp peak at 40° ^14^. Upon annealing in ammonia, the peaks are still located at the same angle, however the intensity of the peaks decreased (Fig. 2b,ii). Note that both oxides and oxynitrides are usually having virtually overlapping XRD patterns^9–12^.

As XRD could not distinctively confirm the formation of TiON nor the presence of the Ag nanoparticles, XPS being a surface sensitive technique, is believed to resolve the differences between the oxides and oxynitrides^10^. Figure 3 shows the XPS high-resolution scans of the four elements; Ti, O, N and Ag for air-annealed, ammonia-annealed, and Ag-decorated ammonia-annealed nanotube samples and the data are listed in Table 1. Figure 3a shows the Ti 2p XPS lines. The Ti 2p spectrum of the air-annealed TiO_2_ sample (Fig. 3a,i) exhibits two peaks at 458.5 and 464.3 eV characteristic of Ti 2p_3/2_ and Ti 2p_1/2_, with a spin orbit splitting of 5.8 eV, indicating Ti^4+^ oxidation state^15^. Upon annealing in ammonia (Fig. 3a,ii) and Ag decoration (Fig. 3a,iii), both peaks are shifted from their original positions, (Fig. 3a,ii,iii). The shift to low energy side is a signature of increasing electron cloud density around Ti. This can be related to the introduction of a less electronegative atom into the crystal lattice of TiO_2_. This finding suggests the introduction of N into the titania lattice because it has a smaller electronegativity (3.04 on Pauling scale) compared to O (3.44 on Pauling scale)^28^. Figure 3b shows O1s XPS spectra acquired for air-annealed, ammonia-annealed, and Ag-decorated nanotube samples. The spectrum shown in Fig. 3b,i exhibits small shoulder at 532.2 eV and a singlet peak at 531.4 eV. The peak at 531.4 eV is attributed to O-H groups, and the small shoulder at 532.2 eV can be attributed to physisorbed water^29^. The position of the shoulder shifted a little to lower binding energies after annealing in ammonia (ii, iii). Such shift caused by the increase of titanium in low valence states^29^. Note that the Ti 2p_3/2_ photoemission line at 458.5 eV is diagnostic for oxynitride (Ti-O-N)^29^. Figure 3c shows the N1s XPS spectra acquired for air-annealed, ammonia-annealed, and Ag-decorated nanotube samples. The N 1 s peak observed at 402.3 eV can be attributed either to incorporation of nitrogen into the nanotubes^30, 31^. or to chemisorbed nitrogen^30–32^. Clearly one can see a small shoulder at 400 ± 0.2 eV (Fig. 3c,ii,iii), which can be ascribed to γ-N state, which is molecularly chemisorbed N_2_. Additionally, the peak at 396 ± 0.2 eV belongs to β-N state, which is essentially atomic N in the form of mixed titanium oxide-nitride (TiO_2−x_N_x_). This indicates that the heat treatment in ammonia atmosphere indeed leads to the substitution of some oxygen sites by nitrogen^33^, see Table 2. This finding is in good agreement with previous results on N-doped TiO_2_^17^. Figure 3d is Ag 3d core level XPS scan over a small energy window at higher resolution. The Ag 3d_5/2_ peak appears at 368.3 eV and the Ag 3d_3/2_ peak is found at 374.3 eV, with a splitting of the 3d doublet of 6.0 eV, indicating that Ag mainly exists in metallic state on the sample of Ag-decorated nanotubes^34, 35^.

**Table 1:** Atomic percentage of Ti, O, N and Ag for the air-annealed, ammonia-annealed, and Ag-decorated ammonia-annealed samples as extracted from XPS.

Sample	Ti	O	N	Ag
Air-annealed	21.93	76.86	1.21	—
Ammonia-annealed	23.31	44.62	32.07	—
Ag-decorated ammonia annealed	38.23	54.34	6.87	1.09

**Table 2:** Traditional and Kröger-Vink notations of defects in TiO_2_ and N-doped TiO_2_ systems.

Traditional Notation	Description	Kröger-Vink Notation
Ti_Ti_^+4^	Ti^+4^ ion in titanium lattice site	Ti_Ti_^x^
Ti_Ti_^+3^	Ti^+3^ ion in titanium lattice site	e’
V_Ti_	Titanium vacancy	V_Ti_””
Ti_i_^+3^	Ti^+3^ in an interstitial site	Ti_i_^•••^
Ti_i_^+4^	Ti^+4^ in an interstitial site	Ti_i_^••••^
O_O_^−2^	O^−2^ ion in an oxygen lattice site	O_O_^x^
V_O_	Oxygen vacancy	V_O_^••^
O_O_^−^	O^−^ ion in an oxygen lattice site	h^•^
N_O_^−3^	N^−3^ ion in an oxygen lattice site	N_O_”’
N_i_^−3^	N^−3^ ion in an interstitial site	N_i_^•••^

**Figure 4:**
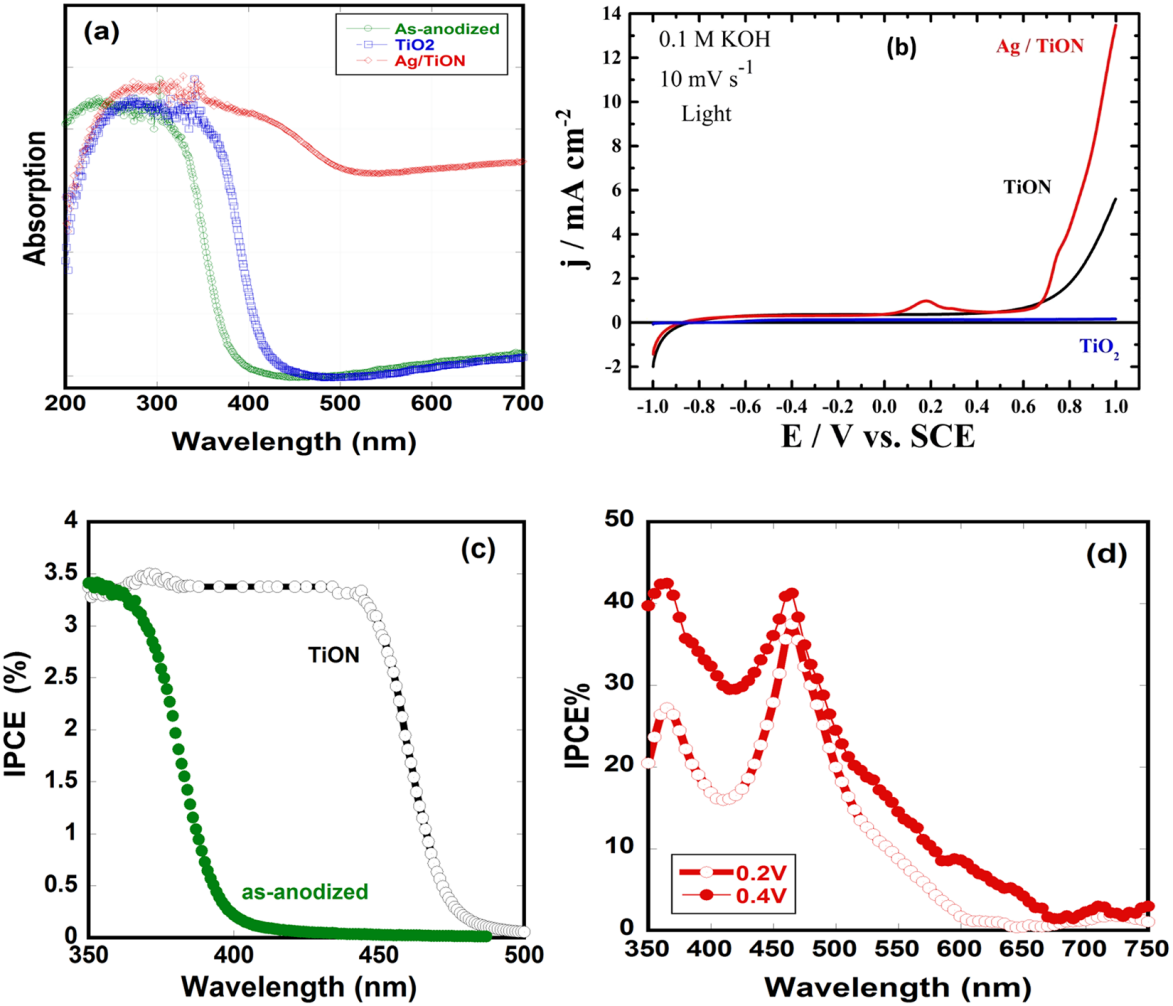
(**a**) UV-Vis absorption spectra of as-anodized nanotubes, TiO_2_ nanotubes annealed in air and Ag-decorated TiON nanotubes(Ag/TiON), (**b**) linear sweep voltammetry under illumination of TiO_2_, TiON and Ag/TiON, (**c**) the IPCE under no bias of as-anodized and TiON samples, and (**d**) the IPCE of Ag/TiON under applied bias.

Figure 4a shows the UV-Vis absorption spectra of as-anodized, air-annealed and Ag/ammonia-annealed nanotube (Ag/TiON) samples. Annealing in air resulted in a small red-shift in the absorption wavelength from 385 nm (3.2 eV) to 410 nm (3.03 eV). However, annealing in ammonia resulted in a significant red-shift in the visible region up to 512 nm (2.4 eV). Note also the hump at 430 nm, which could be related to the presence of Ag nanoparticles. This is in agreement with Ferrero *et al*.^15^ who showed that titanium oxynitride mesoporous thin films are efficient visible-light-active photocatalysts due to the discrete introduction of N, which caused a shift of the titania absorption edge. The photocatalytic activity of the fabricated Ag/TiON nanotubes was investigated by using them as photoanodes to split water under AM 1.5 G one-sun illumination. Figure 4b shows the photocurrent density of the air-annealed, oxynitride, and Ag/oxynitride nanotube electrodes. The photocurrent produced by the air-annealed nanotube (0.15 mA/cm^2^ at 1.0 V_SCE_) is found to be in agreement with those reported in the literature^36^, indicting the high quality of the nanotubes. Interestingly, the oxynitride nanotubes showed exceptional enhancement in the photocurrent density (6 mA/cm^2^ at 1.0 V_SCE_) compared to the air-annealed nanotubes and also compared to that reported for TiN nanostructured thin film (0.2 mA/cm^2^ at 1.0 V_Ag/AgCl_)^37^. Such an enhancement is in accordance with the absorption spectra shown in Fig. 4a. Upon addition of the Ag nanoparticles to the oxynitride nanotubes, the photocurrent significantly increased to 14 mA/cm^2^ at 1.0 V_SCE_. Such enhancement can be relatd to increasing the conductivity and the possible plasmonic effect of Ag nanoparticles. This is in agreement with the onset potential, the light contribution toward the minimum potential needed for water splitting process to take place, as it is shifted to more negative values in the order: TiO_2_ (−0.749 V_SCE_) < TiON (−0.84 V_SCE_) < Ag/TiON (−0.961 V_SCE_). Therefore, the Ag/TiON nanotubes photoanode requires less voltage for water oxidation than the TiO_2_ and TiON nanotube photoanodes counterparts, indicating more favorable photoelectrochemical activity.

The incident photon conversion efficiency (IPCE) experiments were performed in a two-electrode arrangement with the TiO_2_, TiON, or Ag/TiON nanotube array films as the working photoelectrodes and platinum foil as a counter electrode in 0.1 M KOH solution. Figure 4c shows the obtained IPCE for the nanotube array films as a function of the irradiation wavelength under no bias. The IPCE was calculated using Eq. 1, where λ is the wavelength of incident light, *i*_*ph*_ is the photocurrent density under illumination at λ and *I*_*o*_ is the incident light intensity at λ.

1 $${IPCE} \% =\,\frac{(1240\,eV.nm)\times ({i}_{ph}mA.c{m}^{-2})}{({\lambda }\,nm)\times ({I}_{o}\,mW.c{m}^{-2})}\times 100$$

The obtained IPCE values, Fig. 4c, in the wavelength range from 400 to 550 nm indicate the activity of TiON films in the visible light, in accordance with the absorption spectra shown in Fig. 4a. Note that the Ag/TiON films showed similar IPCE behavior except for a small hump at 480 nm. The applied bias assists the separation of the photogenerated electron-hole pairs, thereby enhancing the IPCE. Upon the use of 0.2 V and 0.4 V (Fig. 4d), Ag/TiON films showed an enhancement in the IPCE: between 350–400 nm, the IPCE increases up to 25%, then it further increases to 41% in the wavelength range 450–510 nm, after which it declines indicating that the photocurrent occurs as a result of the band gap transition. Note that the maximum IPCE peak was observed around 480 nm, which is the commonly reported plasmonic peak or Ag nanoparticles^38^, suggesting that the enhancement in the photoelectrochemical activity is partially supported by the plasmonic effect of Ag NPs. The obtained IPCE for TiON and Ag/TiON nanotube films are much higher than that obtained for the pristine TiO_2_ nanotube film, in good agreement with the UV-vis DRS results shown in Fig. 4a. We note that our obtained IPCE is higher than that reported for N-doped titanium dioxide nanotube arrays^39^.

Considering the correlation between the structure of the fabricated photoanodes and the observed enhanced photoresponse, the thin wall thickness of the synthesized TiON nanotube arrays is expected to play a vital role. The nanotubular architecture, with a wall thickness of 14 ± 2 nm, ensures that the photogenerated holes are never generated far from the semiconductorelectrolyte interface^40^. Furthermore, since half the wall thickness is significantly less than the minority carrier diffusion length (~20 nm in TiO_2_)^41^, charge-carrier separation takes place efficiently. The potential drop (${\rm{\Delta }}{\varnothing }_{0}$) within the tube wall was shown to follow the relation^36^:

2 $${\rm{\Delta }}{\varnothing }_{0}=\,\frac{kT{r}_{0}^{2}}{6e{L}_{D}^{2}}$$

where *r*_*0*_ is half the width of the wall, T is the temperature, and L_D_ is the Debye length, given by^42^:

3 $${{\rm{L}}}_{{\rm{D}}}={[\frac{{{\rm{\varepsilon }}{\rm{\varepsilon }}}_{0}{\rm{kT}}}{2{{\rm{e}}}^{2}{{\rm{N}}}_{{\rm{D}}}}]}^{2}$$

where N_D_ is the number of ionized donors per cubic centimeter^42^. It is important to note that this potential drop across the wall thickness may not be enough to separate the photogenerated electrons and holes. However, because of the nanoscale dimensions of the walls, the holes can easily diffuse into the surface, which was shown to takes place on a scale of picoseconds^43^. It was also reported that minority carriers generated within a distance from the surface equal to the sum of the depletion layer width and the diffusion length (retrieval length) escape recombination and reach the electrolyte^44^. Note that the relevant dimensional features of our TiON nanotube arrays (half the wall thickness) are all smaller than 10 nm, which is the range reported for crystalline TiO_2_ retrieval length^45^. Therefore, bulk recombination is expected to be reduced and the photoconversion efficiency to be enhanced^46–48^.

## Conclusions

In summary, we report the first demonstration of a facile method for the fabrication of highly ordered titanium oxynitride nanotubes with large surface area and high crystallinity. The as-anodized TiO_2_ array films retain their morphology upon annealing in ammonia ambient, realizing the opportunity to convert TiO_2_ into TiON at temperatures as low as 600 °C. Interestingly, titanium oxynitride nanotubes showed significant increase in the photocurrent (6 mA/cm^2^) compared to the as-anodized TiO_2_ nanotubes counterpart (0.15 mA/cm^2^). In addition, decorating the TiON nanotubes with Ag nanoparticles resulted in exceptionally high photocurrent reaching 14 mA/cm^2^ at 1.0 V_SCE_. Finally, this proposed platform of titanium oxynitride nanotubes array films holds promise for a variety of applications of the future design of optoelectronic devices.
